# To Hit or Not to Hit, That Is the Question – Genome-wide Structure-Based Druggability Predictions for *Pseudomonas aeruginosa* Proteins

**DOI:** 10.1371/journal.pone.0137279

**Published:** 2015-09-11

**Authors:** Aurijit Sarkar, Ruth Brenk

**Affiliations:** 1 Division of Biological Chemistry & Drug Discovery, College of Life Sciences, University of Dundee, Dow Street, Dundee, United Kingdom; 2 Institut für Pharmazie und Biochemie, Johannes Gutenberg-Universität Mainz, Mainz, Germany; 3 University of Bergen, Department for Biomedicine, Bergen, Norway; Universite de Sherbrooke, CANADA

## Abstract

*Pseudomonas aeruginosa* is a Gram-negative bacterium known to cause opportunistic infections in immune-compromised or immunosuppressed individuals that often prove fatal. New drugs to combat this organism are therefore sought after. To this end, we subjected the gene products of predicted perturbative genes to structure-based druggability predictions using DrugPred. Making this approach suitable for large-scale predictions required the introduction of new methods for calculation of descriptors, development of a workflow to identify suitable pockets in homologous proteins and establishment of criteria to obtain valid druggability predictions based on homologs. We were able to identify 29 perturbative proteins of *P*. *aeruginosa* that may contain druggable pockets, including some of them with no or no drug-like inhibitors deposited in ChEMBL. These proteins form promising novel targets for drug discovery against *P*. *aeruginosa*.

## Introduction

Attrition rates in the drug discovery process are high. Studies have revealed that 80% of drug discovery projects fail to produce clinical candidates and only 2% actually produce marketed drugs. Poor target selection was found to be a major factor for the failures [1]. Hence, target selection is an important consideration for drug discovery. The advent of large-scale genomics projects has introduced a plethora of plausible targets. Target selection has traditionally been guided by biological or technical aspects such as assay feasibility. However, high-throughput screening against targets where chemical tractability has not been established may lead to unsatisfying hit rates [2]. This raises the need for low-cost methodologies to assist with target selection. Recently, computational methods have been introduced for this purpose. One option is to prioritize targets based on homology with other targets already possessing high-affinity ligands [3–7]. Alternatively, one can assess the binding sites of potential targets to estimate their suitability as drug targets.

Targets likely to bind orally bioavailable drugs with high affinity possess binding sites that complement the nature of these molecules These binding sites are commonly referred to as being druggable [8]. This distinguishes them from other binding sites which are referred to as “less-druggable” or “non-druggable”. The probability of deriving a drug-like ligand with high binding affinity for a protein possessing a “druggable” pocket is therefore higher than for others. The term “druggability” itself is hotly debated and several alternative terms such as “bindability”, “ligandability”, “tractability” or “chemical tractability” have been proposed. We will use the term “druggability” throughout this manuscript because it is the prevalent term used in literature.

Over the last few years, several methods have been reported that are able to segregate druggable pockets from less-druggable ones [9–20]. DrugPred is one such druggability prediction method [21]. DrugPred describes the size and shape of the binding site using a “superligand”, which is obtained by merging predicted binding modes of drug molecules that were docked into the pocket using only steric constraints. Descriptors encoding polarity and size of the pocket are subsequently calculated based on the superligand and used to predict the druggability of the binding site. We had previously found DrugPred to perform uniformly on the NRDLD dataset containing proteins with druggable and less druggable binding sites as well as other datasets, with ~90% prediction accuracy, which was superior to other methods we had tested [21]. Desaphy *et al*. found that our linear model was approximately as accurate as their Support Vector Machine-based method, both of which were better than two other methods [11]. Even though the variability in metrics used to determine robustness of druggability prediction methods makes it difficult to meaningfully compare and contrast them, it is clear that DrugPred is one of the more accurate and reliable methods reported thus far [8,11]. Here, we test the use of this method in identifying druggable proteins on a genome-wide scale. For this study, we chose *Pseudomonas aeruginosa* as a model organism.

*P*. *aeruginosa* is a Gram-gram negative bacterium that has proven to be difficult to treat with antibiotics. It often causes opportunistic infections in hospitalized patients of cystic fibrosis [22] and burn victim who are immunosuppressed or immunocompromised [23]. Chemotherapeutic intervention is therefore required, which is made difficult when infection is caused by resistant strains of bacteria. Studies with transposon mutant libraries have identified perturbative proteins in *P*. *aeruginosa*, i.e. proteins that are either essential, potentially essential or else virulence factors [24,25]. A comprehensive database of *P*. *aeruginosa* genes and related information is available in the AEROPATH database (aeropath.lifesci.dundee.ac.uk), including essentiality labels as described by the above studies [24,25]. There are 5677 genes reported in the AEROPATH database, of which 992 are predicted to be perturbative. Crystal structures are available in the public domain (RCSB Protein Data Bank) for 77 of the perturbative gene products. Crystal structures are also available for homologs of 565 of the remaining perturbative proteins.

Structures of perturbative genes in the AEROPATH database were analysed using DrugPred in order to evaluate the use of such methods for genome-wide druggability predictions and to prioritize proteins for drug discovery. While it was straightforward to assess pockets of available crystal structures of *P*. *aeruginosa* proteins, the real challenge was to make predictions for pockets in proteins where no solved structure was available. To this end, we established a work flow for homology-based druggability assessment. We also compared the predictions to chemogenomics-based predictions and discuss similarities between the two systems, along with the advantage of using both systems simultaneously in order to prioritize targets. Finally, we suggest potential new drug targets for *P*. *aeruginosa*.

## Results

### Constructing DrugPred 2.0

Analysis of protein druggability on a large scale required the use of a high-throughput druggability prediction algorithm. In order to scale up the original DrugPred’s performance, we re-engineered the method for smooth execution on a compute cluster and introduced some changes in the descriptor calculation methodology. Previously, the relative polar surface area (psa_r), total hydrophobic surface area (hsa_t) and total contact surface area (csa) of a binding site were calculated using the MOLCAD module in SYBYL-X (Tripos, St. Louis, Missouri, USA) [21]. In the current version, these descriptors were calculated with Openeye’s OEChem TK and Spicoli TK (OpenEye Scientific Software, Santa Fe, NM) [26,27]. This decision was made due to license restrictions and easier integration in the Python code. All other descriptors were calculated as reported previously [21]. Retraining and revalidation of the original DrugPred statistical models was carried out to identify any changes in performance. The NRDLD dataset of 115 structures was modified as per previous recommendations [21]. Thiamine purophosphokinase (TPK) was eliminated because there are no clinically used drugs for this target, apart from thiamine, which is actually a substrate, making it unclear if TPK is druggable. Urokinase Plasminogen activator (uPA) and human thymidine phosphorylase (HTP) were eliminated, because all known modulators are either highly charged or else require administration as prodrugs, against the very definition of druggability [21]. The binding sites of hydroxynitrile lyase (HNL) and angiotensin-converting enzyme 1 (ACE-1) vary significantly from other members of the dataset, making them unsuitable for model development [21]. Thereafter, the new dataset containing 110 proteins was divided into a training set of 75 structures and a validation set of 35 structures (Table A in S1 File). Descriptors were calculated for the training set and a new Partial Least Squares-Discriminant Analysis (PLS-DA) model was built as done previously [21]. The new model, which we named DrugPred 2.0, was used to obtain scores for all training and test set structures, following which accuracy, precision and recall values were calculated as done previously [21]. Briefly, accuracy describes the success rate of categorizing pockets as druggable or less-druggable, precision describes the rate of correct calls by the model (e.g. how many data points predicted to be druggable were truly druggable), and recall describes the ability of the model to correctly identify members of a category (e.g., how many of the druggable proteins were correctly categorized). Comparing the two models, we found that all three statistical values were similar for both models. In our original report, we had also employed an ambiguous zone. This is a region of scores where it is difficult to identify targets as belonging to either the druggable or less-druggable categories [21]. When applying such a zone with DrugPred 2.0, similar results were obtained (Table 1). For the sake of simplification, during the rest of this manuscript, we will highlight only those results where an ambiguous zone was employed and any data points that lay within this zone were discarded for calculation of final results.

**Table 1:** Accuracy, recall and precision values for training and validation sets for DrugPred 1 and 2.0.

DrugPred version	Data set	With/ without ambiguous zone	Accuracy	Recall (Druggable/ Less druggable)	Precision (Druggable/Less druggable)
1 **^Ω^**	**Training**	Without	0.91	0.96/0.83	0.90/0.93
2.0			0.91	0.94/0.86	0.92/0.89
1 **^Ω^**		With	0.92	0.95/0.86	0.91/0.93
2.0			0.95	0.98/0.9	0.95/0.95
1 **^Ω^**	**Validation**	Without	0.89	0.91/0.85	0.91/0.85
2.0			0.94	0.95/0.93	0.95/0.93
1 **^Ω^**		With	0.91	0.91/0.92	0.95/0.86
2.0			0.97	1.00/0.92	0.95/1.00

^**Ω**^ Values taken from Krasowski et al [21]

### Homolog-based druggability predictions

For the majority of essential or potentially essential proteins from *P*. *aeruginosa* in the AEROPATH database no crystal structure was deposited in the PDB. However, structures of homologous proteins were available for 565 of them. It is common practice to assume that homologs of a target already known to be modulated by small molecules are druggable as well, particularly if the sequence homology is high [3–7,17]. It was therefore interesting to test whether DrugPred predictions could be transferred between homologous pockets as well. We also wanted to establish a sequence identity cut-off at which such transfers could be made and a minimum number of structures required for reliable transfers.

**Fig 1:**
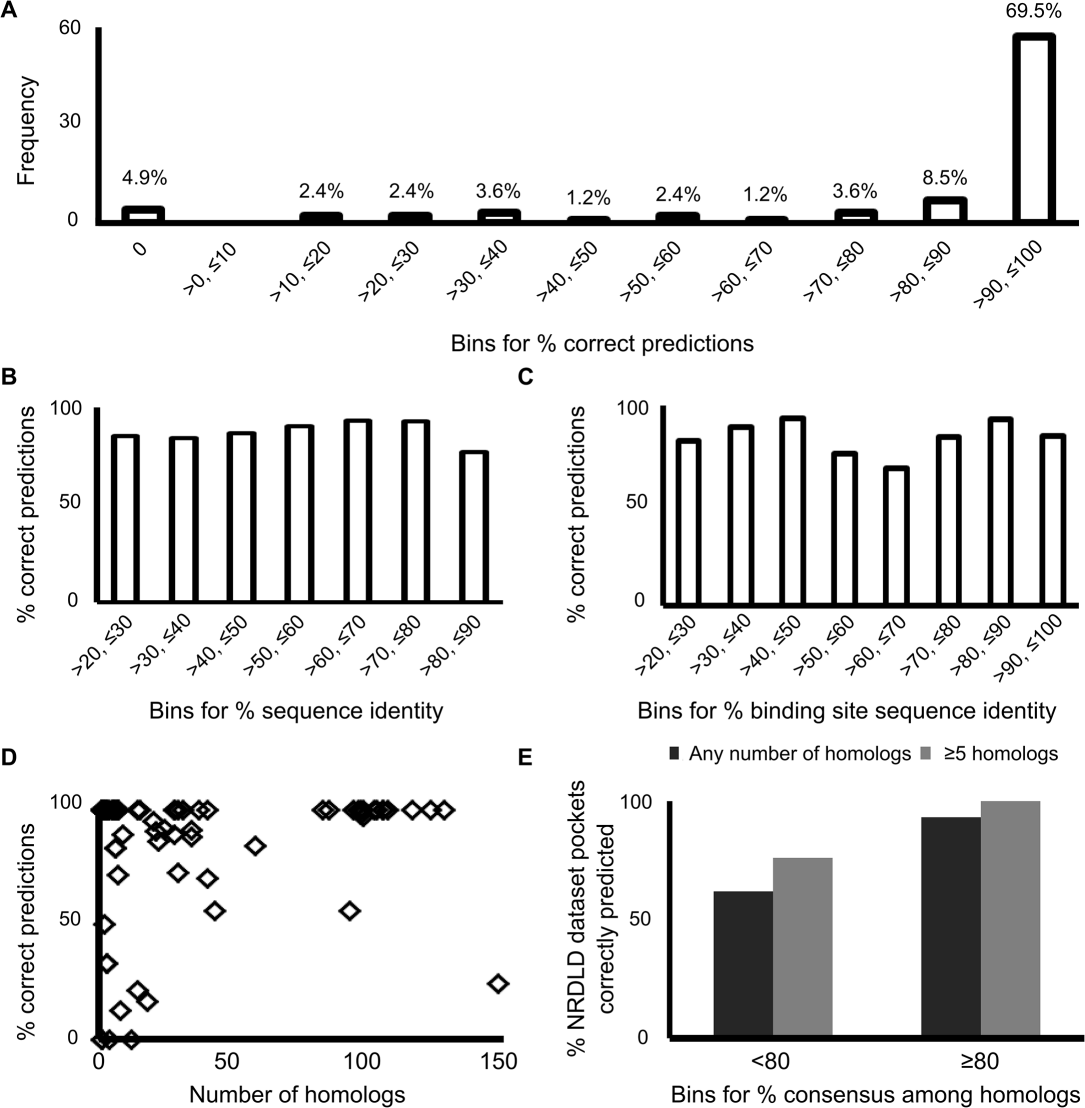
The basis for homolog-based druggability predictions. (A) Homologous pockets whose classification correctly reflected the druggability of the parent pocket. The data was binned according to percent correct predictions among the scored pockets for each parent homolog. The number of NRDLD proteins that fitted into each category was then plotted (frequency and percentages are both shown). (B) Correct predictions in relationship to sequence identity. The percent identity between NRDLD dataset structures and homologous chains was noted. The homologs were then binned according to their percent sequence identity. The percent of homologs whose predictions matched that of the NRDLD dataset pocket was plotted for each bin. (C) Correct predictions in relationship to sequence identity of binding site residues only. Plotted as described for (B), but instead of the sequence identity only the identity of the binding site residues was used. (D) Percent correct predictions in relationship to number of assessed homologs. (E) Percent consensus in relationship to percentage of correctly predicted NRDLD dataset pockets. The NRDLD dataset pockets were binned into two categories, where either <80% or ≥80% consensus (see methods) in druggability predictions for their respective homologs was observed. The percentage of NRDLD pockets whose druggability was correctly reflected by consensus amongst their homologs was then plotted for each of these bins.

With this aim in mind, we embarked on a study to identify structural homologs of the modified NRDLD dataset and to score their pockets using DrugPred 2.0. The predictions were then compared to the classification of the parent structures. Homologous structures were found for all but three proteins in the dataset. For 19 proteins, none of the homologous structures contained a ligand to mark the binding site and they were therefore not considered further. The druggability of the homologous binding sites in the remaining 88 proteins was predicted. The predictions for all homologs of six of these proteins were outside the model as judged by high distance-to-model in X-plane (DModX) values. DModX represents the distance of a data point from a hyperplane that represents the model. Smaller values demonstrate a higher likelihood that data points are within the predictive domain of the model, while higher values demonstrate that predictions for the data points may be unreliable. Predictions with a high DModX value were therefore not analysed further. Thus, the final dataset consisted of 3186 homologous pockets for 82 proteins. The total number of homologous pockets per dataset pocket ranged from 1 to 208 and the sequence identity between the homologs and parent proteins from 22.3 to 89.9% (Table B in S1 File). The percentage of homologous pockets whose classification correctly reflected the druggability of the parent pocket ranged from 0% to 100% (Fig 1A). In the majority of the cases the druggability prediction of the homologous pockets was correctly transferable to the parent pocket, e.g. for 57 out of 82 proteins at least 90% of the homologous pockets provided the correct prediction. Of these 57, 51 showed 100% correct predictions. However, there were 12 instances where more than 50% of predictions for homologs did not match the druggability of the dataset pocket. Therefore, we attempted to identify filter criteria to obtain more reliable predictions.

It would be useful if we could establish (A) a sequence-cut off, (B) a requirement of minimum number of homologs, and/or (C) a minimum percent consensus in predictions for homologs, as suitable criteria to make the predictions more reliable. Hence, we first plotted the percent homologs that reflected the parent structure’s druggability (% correct predictions) for various bins of percent sequence identity to the parent structure (Fig 1B). There was no relationship between sequence identity of the homologous proteins and the percentage of correct predictions observed. The same was observed when instead of the sequence identity only the identity of the binding site residues was considered (Fig 1C). Similarly, percent correct predictions did not increase with the number of homologs used for assessment (Fig 1D). We went on to investigate if the parent protein pocket’s druggability could be predicted by a consensus between its homologs. For this purpose, percent consensus between different homologous pockets belonging to the same NRDLD parent entry was calculated using the formula: (100*|#druggable—#less-druggable|)/(total number of predictions). This measure enabled us to identify how many homologous pockets gave us the same prediction regardless of the prediction itself. We then binned the NRDLD pockets into two categories, with either ≥80% consensus or <80% consensus amongst all homologs (Fig 1E). With <80% consensus in their druggability prediction, only ~62% of the parent NRDLD pocket’s druggabilities was predicted correctly. However, with at least 80% consensus, ~93% of the parent NRDLD pockets were correctly predicted. The latter value increased to 100%, when additionally at least 5 homologs were assessed. Hence, we identified a dual-filter approach to obtain reliable homolog-based druggability predictions using DrugPred 2.0. This formed the basis for the subsequent evaluation of *P aeruginosa* proteins where no crystal structure has yet been solved.

### Structure-based druggability analysis of *P*. *aeruginosa* proteins

Information for 5677 genes is stored in the AEROPATH database (Table 2). Of those, 992 are annotated to be perturbative. Crystal structures for 77 of those gene products have been determined. Homologous structures for a further 565 perturbative gene products were available. The available crystal structures were subjected to structure-based druggability predictions using DrugPred 2.0.

**Table 2:** Overview of triaging the genes stored in the AEROPATH database to enable structure-based druggability predictions for *P*. *aeruginosa* proteins.

	Number of entities
1) Genes stored in AEROPATH database	5677
2) Annotated to be perturbative	992
3) Perturbative gene products originating from *P*. *aeruginosa* for which crystal structures are available	77
3a) Proteins for which valid druggability predictions were obtained	24
3b) Proteins predicted to be druggable (Table 3)	13
4) Proteins encoded by perturbative genes for which crystal structures of homologous proteins were available	565
4a) Proteins for which valid druggability predictions were obtained	241
4b) Proteins predicted to be druggable (Table 4)	16

The analysis of available *Pseudomonas* protein structures was straightforward. Pockets were identified by bound ligands. Subsequently, the druggability was assessed using DrugPred 2.0 and pockets not fitting to the model were removed (as judged by DModX values). A gene product was considered druggable if it possessed at least one pocket with a druggable score. Following this procedure druggability scores could be obtained for 103 pockets from 34 *Pseudomonas* proteins (out of 77 perturbative genes for which crystal structures were available, Table 2). Of those 13 were found to possess pockets likely to bind drug-like molecules with high affinity (Table 3).

**Table 3:** List of *P*. *aeruginosa* protein crystal structures containing pockets that are predicted to be druggable.

Pseudomonas gene code	Product name	Gene name	PDB code	Ligand	Chemogenomics-based druggability rank^a^
PA0019	Polypeptide deformylase	def	1lry	BB2	3 (2)
			1ix1	BB2	
PA0395	Twitching motility protein	pilT	3jvv	ACP	-
PA1148	Exotoxin A precursor	toxA	1aer	AMP	-
			1dma	AMP	
				NCA	
			1aer	TIA	
			1xk9	P34	
			1zm9	P34	
PA1430	Transcriptional regulator	lasR	2uv0	OHN	8 (6)
			3ix3	OHN	
			3ix4	TX1	
			3jpu	TY4	
			3ix8	TX3	
PA1900	Probable phenazine biosynthesis protein	phzB2	3ff0	UNL	-
PA2386	L-ornithine-N5-oxygenase	pvdA	3s61	ORN	-
PA3155	UDP-2-acetamido-2-dideoxy-D-ribohex-3-uluronic acid transaminase	wbpE	3nyu	LLP	-
PA3540	GDP-mannose-6-dehydrogenase	algD	1muu	GDX	-
			1mv8	GDX	
PA3724	Elastase	lasB	3dbk	RDF	4 (3)
PA4279	Hypothetical protein (probable pantothenate kinase)		2f9w	PAU	-
PA4406	UDP-3-O-acyl-N-acetylglucosamine deacetylase (LpxC)	lpxC	2ves	GVR	1
PA4407	Cell division protein	ftsZ	1ofu	GDP	115 (29)
PA5163	Glucose-1-phosphate thymidylyltransferase (RmlA)	rmlA	1g3l	TRH	-
			1fxo	TMP	

^a^Ranks obtained when only perturbative proteins are considered are given in brackets.

**Fig 2:**
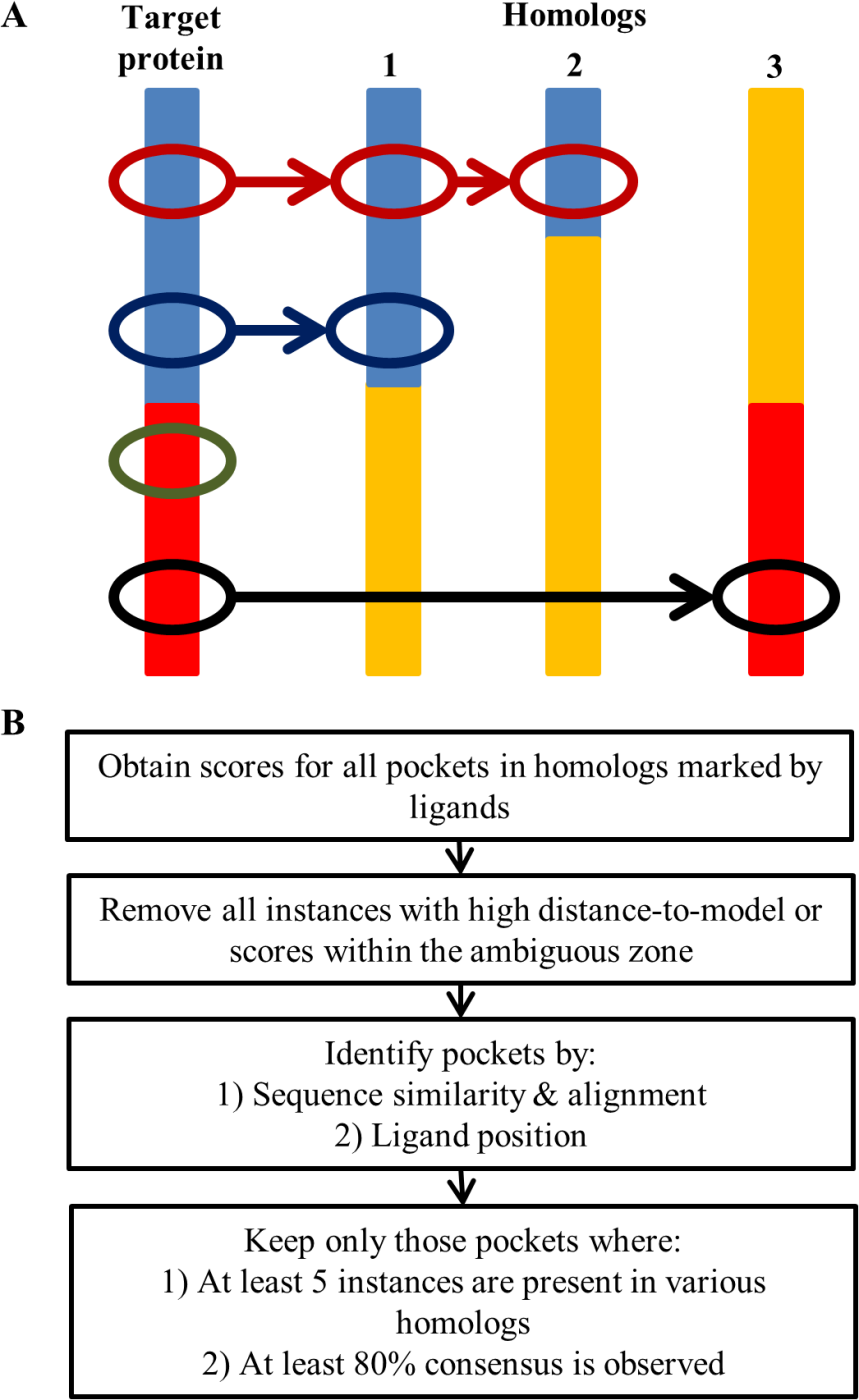
Complexity of homolog-based identification of pertinent pockets in proteins. A) A hypothetical target protein is depicted with three homologous proteins. The target protein consists of two domains, one shown in blue and the other in red. These domains may be represented by complete or partial sequences in homologs. For example, homologs 1 and 2 possess short domains homologous to the target protein's blue domain. On the other hand, homolog 3 possesses a sequence match for the red domain. Each target protein domain possesses pockets (denoted by black, green, red and blue ovoids), which may or may not be identified by the presence of ligands in homologs. Here, the black pocket is also represented in homolog 3, but the green pocket is not. The red pocket is observed in both, homolog 1 and 2, but the blue pocket is only represented in homolog 1. B) Workflow to identify druggable pockets in homologs proteins.

The analysis of gene products lacking crystal structures, but where homologous structures were available, was more convoluted. In the AEROPATH database crystal structures of homologous proteins are linked to *P*. *aeruginosa* gene products. However, there is no annotation if these crystal structures cover the same sequence segment of the gene, which is particularly relevant for multi-domain and multi-pocket proteins (Fig 2A). Therefore, it had to be ensured that only corresponding pockets of the homologs were compared when assessing the druggability of a parent protein. This was achieved using the following workflow (Fig 2B): First, DrugPred 2.0 was used to score all pockets marked by a ligand in the structures of homologous proteins for a particular parent sequence. In the next step any pockets with high DModX values or scores within the ambiguous zone were discarded. Subsequently, the sequence of residues forming each homologous pocket was determined. Next, the domains represented by this sequence was determined by pairwise BLAST [28] searches with an E-value cut-off of 1e^-5^. For each domain identified, the homologous structures were aligned and the number of pockets represented was then determined by pairwise comparison of ligand positions. For final assessment, only corresponding pockets represented in at least five different crystal structures were retained. A protein pocket was considered to be druggable, if at least 80% consensus was reached among the homologs and the majority of the pockets were predicted druggable. Following this workflow, starting from 565 gene products for which homologous structures were available, valid (low DModX) predictions were obtained for 241 (Table 3). A total of 16 *P*. *aeruginosa* gene products were found to possess pockets likely to bind drug-like ligands (Table 4).

**Table 4:** List of *P*. *aeruginosa* proteins predicted to possess a druggable pocket.

PA Code	Representative homolog (PDB code / ligand)	# of HA^α^	% Sequence ID range	# of predictions druggable / less druggable / ambiguous	Product Name	Gene Name	Rank^Ω^
PA0350	2qk8 / MTX	11	39.76–48.78	11 / 0 / 0	Dihydrofolate reductase (DHFR)	folA	85 (22)
PA0363	1b6t / COD	7	40.51–60.00	6 / 1 / 0	Phosphopante-theine adenylyltrans-ferase (PPAT)	coaD	-
PA1648	2dm6 / IMN	6	38.07–39.66	6 / 0 / 0	Probable oxidoreductase		685 (126)
PA1671	1ig1 / ANP	9	26.85–28.66	9 / 0 / 0	Serine-threonine kinase Stk1	Stk1	195 (42)
PA1778	1cbf / SAH	9	28.65–46.15	9 / 0 / 0	Uroporphyrin-III C-methyltrans-ferase	cobA	-
PA2086	1vj5 / CIU	7	29.82–29.82	7 / 0 / 0	Probable epoxide hydrolase		336 (62)
PA2344	1tz3 / AIS	6	25.10–30.35	5 / 0 / 1	Fructokinase	mtlZ	-
PA2965	1b3n / CER	9	39.49–66.50	9 / 0 / 0	Beta-ketoacyl carrier protein synthase II	fabF1	119 (30)
PA2967	1doh / NID	8	34.23–37.70	7 / 0 / 1	3-oxoacyl-[acyl carrier protein] reductase	fabG	80 (21)
PA3883	1a27 / EST	9	34.81–35.19	9 / 0 / 0	Probable short-chain dehydrogenase		32 (11)
PA4068	1lrl / UPG	6	30.97–33.55	6 / 0 / 0	Probable epimerase		544 (98)
PA4385	1a6e / ADP	17	21.93–80.69	17 / 0 / 0	GroEL protein	groEL	-
PA4386	1aon / ADP	7	57.45–61.46	7 / 0 / 0	GroES protein	groES	-
PA4439	1i6k / TYM	11	31.66–33.73	9 / 0 / 2	Tryptophanyl-tRNA synthetase	trpS	-
PA5174	1b3n / CER	8	27.88–28.36	8 / 0 / 0	Probable beta-ketoacyl synthase		488 (83)
PA5288	1v3s / ATP	12	41.07–77.68	10 / 1 / 1	Nitrogen regulatory protein P-II 2	glnK	-

^**α**^ ***HA*:** distinct homologous pockets assessed

^Ω^ ***Rank*:** Chemogenomics-based druggability rank. Ranks obtained when only perturbative proteins are considered are given in brackets.

### Experimental support for DrugPred-based findings

It is already known that some proteins, out of those predicted to be druggable using DrugPred 2.0, are capable of strongly binding drug-like molecules; this is strong support for the predictions. For example, UDP-3-O-acyl-N-acetylglucosamine deacetylase (LpxC, Table 3) is a well-studied target for antibacterial compounds and a number of drug-like ligands has been discovered [29]. Similar, dihydrofolate reductase (DHFR, Table 4) is an established target for antibiotics which are routinely used in man [30]. Recently, drug-like inhibitors of phosphopantetheine adenylyltransferase (PPAT) were discovered and their activity against a range of bacterial strains was demonstrated [31]. An interesting case is also glucose-1-phosphate thymidylyltransferase (RmlA, Table 3). The crystal structure of RmlA was reported earlier [32] (RCSB PDB code: 1fxo), with TMP bound at the active and allosteric sites. Both these sites were assessed using DrugPred 2.0 and it was found that the allosteric site scored druggable while the active site scored less druggable. Interestingly, a high-throughput screen, followed by rational drug design resulted in nanomolar inhibitors of RmlA [33]. These inhibitors were characterised crystallographically and it was observed that all these compounds bound at the allosteric regulatory site that scored druggable.

### Comparison with a chemogenomics-based druggability rankings

Bickerton et al. have introduced a chemogenomics-based druggability prediction method for *P*. *aeruginosa* proteins, which is available through the AEROPATH database. This method utilizes ChEMBL [34] data to identify compounds possessing high affinity or ligand efficiency for specific targets, which are further quantified by Quantitative Estimate of Druggability (QED) measurements [35]. Proteins with a larger number of QED-positive compounds are judged to be more druggable. Furthermore, homologs of druggable protein domains are also considered to be druggable. The *P*. *aeruginosa* proteins were then ranked according to the scores obtained (http://aeropath.lifesci.dundee.ac.uk/pages/background).

We compared DrugPred 2.0 predictions with this chemogenomics-based ranking system. Predictions could be obtained for 158 perturbative gene products using the latter system, and for 265 gene products using DrugPred 2.0 (Table 2). There was an overlap of 38 gene products using both methods, 17 of which were predicted to possess druggable pockets by DrugPred. A good agreement was found between DrugPred 2.0 predictions and top scoring chemogenomics-based predictions. All gene products for which crystal structures from *P*. *aeruginosa* proteins were available and which ranked among the top 10 perturbative gene products using the chemogenomics-based system were predicted to be druggable by DrugPred 2.0 (Table 3). Some gene products that ranked low in the chemogenomics-based method were still classified to be druggable by DrugPred 2.0 (Table 4).

## Discussion

The DrugPred methodology was redevised for high-throughput operation, involving the introduction of robust methods for calculation of descriptors. The new version of this druggability prediction method showed accuracy, precision and recall parameters similar to the older version (Table 1) and it is therefore suitable for large-scale predictions.

Criteria for homolog-based druggability predictions were established. Coverage of perturbative *P*. *aeruginosa* gene products by crystal structures is low (Table 2). Crystal structures for only 77 out of 992 perturbable gene products were available. Homolog-based predictions can be used in order to enhance coverage of the genome. It is commonplace to assume that homologs of druggable proteins will be druggable as well [3–7]. Yet, our analysis of a data set containing crystal structures of homologous proteins for the NRDLD dataset does not support such assumptions (Fig 1 and Table B in S1 File). Similar successful predictions were observed for homologs with sequence identities ranging between 20–90%. However, when at least five instances of homologous pockets were present with at least 80% consensus in druggability predictions, 100% correct predictions were obtained (Fig 1E). While the exact numbers will be data set dependent, this provides guidelines on which criteria to apply when transferring structure-based druggability predictions between homologous proteins.

Full coverage of all perturbative *P*. *aeruginosa* proteins could not be achieved despite including homolog-based druggability predictions (Table 2). The main reason is the lack of structural data. While there are 992 perturbative proteins in the *P*. *aeruginosa* proteome, only 77 of them have solved structures. This number increases to 642 when structures of homologs are also considered. However, as more than one crystal structure of a homologous protein is needed to obtain reliable predictions using DrugPred, this number is actually smaller. Another reason lies in the difficulty of identifying pertinent pockets for computational analysis. DrugPred accepts as input to mark a binding site also spheres generated by a pocket prediction program such as FPOCKET [21,36]. However, for the current study we opted to only assess binding sites that contained a ligand. This was done for the following two reasons. 1) Many groups have attempted to devise new pocket prediction methods, however, unambiguous identification of binding sites is not yet possible [37–48]. Consequently, including predicted pocked in a large scale study is likely to introduce errors. 2) The apo-structure binding site conformation does not necessarily represent the bound conformation [49,50]. This makes such pockets less useful for predictions. Even so restricting the input structures to holo-structures further limited the number of proteins we could address we believe that the obtained predictions are more meaningful. It is evident from this discussion that there is a clear need for more structural data to enable better coverage, especially in the presence of ligands. Previous endeavours have been highly successful in this regard and must continue in the future [51].

Druggable proteins were identified by DrugPred 2.0 using crystal structures from *P*. *aeruginosa* proteins itself, or else through its homologs (Table 3 and Table 4). It must be noted that proteins are not necessarily less-druggable *per se* just because a pocket with druggable score was not observed. It’s possible that the crystal structure of the relevant domain or containing a pocket with the required binding site conformation has not yet been crystallized. We therefore consider the druggability status of these proteins as unknown, rather than less-druggable. Some of the predicted druggable proteins like LpxC and DHFR are well known drug targets for antibiotics while others like RmlA and PPAT were only recently pursued and drug-like inhibitors were identified [29,30]. These findings validate our predictions and add confidence that the predictions reported here are reliable. Other proteins were also found to possess druggable binding pockets but have not yet been investigated using small molecules. For 15 out of the 29 druggable proteins, no inhibitors were submitted in ChEMBL to allow a chemogenomics-based ranking. These proteins form promising novel targets for drug discovery against *P*. *aeruginosa*.

The druggability predictions based on chemogenomics-based scoring and made by DrugPred complemented each other. Predictions for 385 perturbative proteins are obtained by combining both methods. The overlap between the methods was rather small with only 38 gene products. The reasons for this are either the lack of crystal structures suitable for druggability predictions (resulting in ranking by only the chemogenomics-based scoring system) or no precedence of drug-like ligands (resulting in predictions only by DrugPred). This clearly demonstrates that a larger coverage can be reached when complementary methods are used. The chemogenomics-based method provides a rank-ordered list but does not provide a direct classification of druggable or less-druggable. Therefore, a direct comparison between both methods is difficult. Still, when looking at the predicted druggable proteins for which crystal structures from *P*. *aeruginosa* were available, a good agreement was observed (Table 3). Low chemogenomics-based ranks for predicted druggable proteins were also observed (Table 4) and can have several reasons: it is possible that the compounds used to derive the chemogenomics-based score target a different binding site than those used for the structure-based predictions. It might also be that the structures used for scoring do not contain the binding site in a conformation relevant for binding a drug-like ligand. Further, there is also possible that drug-like ligands have not yet been described or were not present in the ChEMBL database. Finally, both computational methods have got short-comings and any predictions obtained should always be viewed critically. Nevertheless, assessment by two such independent methods provides confidence in those targets found to be druggable by both and tremendously extends the coverage of the genome. Together, these tools can direct drug discovery efforts.

### Conclusions

The DrugPred methodology was redevised for high-throughput operation, involving the introduction of new methods for calculation of descriptors. The old and new versions of DrugPred showed similar accuracy, precision and recall parameters. Thus, DrugPred 2.0 is suitable for large scale predictions.

A robust workflow and criteria to score homologous protein structures was established to extend genome coverage. This procedure can readily be applied to other organisms for which drug targets are sought.

This work was limited by the availability of solved structures, but with important advances being made all the time in the field of structural biology, such bottlenecks may seize to exist in the not-too-distant future. In the meantime, identification of 29 perturbative proteins of *P*. *aeruginosa* that may contain druggable pockets, including some of them with no or no drug-like inhibitors deposited in ChEMBL, is a remarkable achievement that might drive drug discovery efforts in the right direction.

## Methods

### Construction of DrugPred 2.0

For speed and scale-up reasons, minor changes were introduced to the previous DrugPred version. In particular, Openeye’s OEChem TK (OEChem, version 1.7.4, OpenEye Scientific Software, Inc., Santa Fe, NM, USA) and Spicoli TK (Spicoli, version 1.1.2, OpenEye Scientific Software, Inc., Santa Fe, NM, USA) were used to calculate the relative polar surface area (psa_r), total hydrophobic surface area (hsa_t) and total contact surface area (csa), instead of using Sybyl-X (Tripos, St. Louis, Missouri, USA) as done previously. [26,27] For that purpose, any atoms whose solvent accessibility changed in the presence of the superligand were identified, and classified as polar and hydrophobic, respectively. Their solvent accessible surface in the unbound state (SASA_i_) was then determined and used to calculate the relevant descriptors using the following formulae:

$$\mathrm{csa}=\overset{N}{\underset{i=1}{\sum }}\left({SASA}_{i}\right)$$

hsat=∑i=1N{Ifatomisapolar,(SASAi)else,0

$${psa}_{r}=\frac{csa-{hsa}_{t}}{csa}$$

For building and validating a Partial Least Squares-Discriminant Analysis (PLS-DA) model, the NRDLD dataset was modified as suggested previously and detailed in the result section [21]. The new dataset of 110 structures was thus formulated accordingly. This modified dataset was divided into a training set of 75 structures and a validation set of 35 structures. Descriptors were calculated for the training set and a Partial Least Squares-Discriminant Analysis (PLS-DA) model was built as done previously [21]. The new model was called DrugPred 2.0.

An ambiguous zone for uncertain predictions had previously been defined mathematically using a one-sided 90% cut-off for both categories; 1.28 times the standard deviation of scores for the less druggable structures was added to their mean score. Likewise, the same was subtracted from the mean score for all druggable structures. This exercise generated a region of statistical uncertainty, aptly named the ambiguous zone. DrugPred performed reasonably well outside this zone of uncertainty, but a drop in performance was observed when data points within the ambiguous zone were included. Accordingly, we attempted to establish the ambiguous zone during the construction of DrugPred 2.0 as well. Using the same definition, we found the ambiguous zone to be unreasonably small; suggesting this method for setting boundaries for the ambiguous zone could no longer be used. However, it is reasonable to expect a region of uncertainty with such models, so we arbitrarily built an ambiguous zone with the magnitude of two score units, from 0.4–0.6, which is the middle of the scale and of similar size than the ambiguous zone established with DrugPred 1.0.

Accuracy, precision and recall values were calculated as before [21]. Statistical analysis, including DModX value determination, was carried out in the SIMCA-P+ package (www.umetrics.com/products/simca).

### Preparation of structures for druggability analysis

Ligands were identified in each PDB file and unless a particular ligand was part of the list of cofactors or common additives during the crystallography process (Table C in S1 File), it was treated as a marker for its binding site. Any protein chains, cofactors and metals presenting at least one atom within 3 Å of the ligand were retained as components of the binding site, while the rest were deleted. These reduced PDB files were processed using DrugPred.

### Identification of homologous structures of the NRDLD dataset

The NRDLD dataset, along with ligands marking the binding sites has been reported previously [21] and was modified as described above. The ligand marking the binding site for each dataset structure was identified and the surrounding chain that forms maximum contacts with it was determined. Homologous structures for this chain were identified by sequence abstraction and BLAST searches using a database containing all sequences for structures reported in the RCSB PDB using an E-value cut-off of 1e-5. They were downloaded, followed by structure preparation as detailed above. These structures of homologous proteins were then subjected to structural alignment using PyMOL (The PyMOL Molecular Graphics System, Version 1.2r3pre, Schrödinger, LLC.) and the Openeye OEChem toolkit was used to confirm vicinity of ligands in the homologous chain, confirming that only pockets that are analogous to the one reported in the modified NRDLD dataset were included.

The binding site identity between the parent pocket and the homologous pockets was calculated as follows: 1) A consensus pocket was generated. The consensus pocket consisted of all residues that were either part of the parent pocket or the pocket found in the homolog. For that purpose, the same pocket definition as for the descriptor calculation was used (e.g. change of solvent accessibility in the presence of the superligand). 2) The sequences of the parent protein and the homolog were aligned using ClustalW.[52] 3) For each residue in the consensus pocket it was noted if a sequence match in the alignment was found. The sequence identity of the two binding sites was then calculated as 100*(number of sequence matches)/(number of binding site residues) whereas residues that occurred at the same position in the alignment were only counted once.

Percent consensus between different homologous pockets for the same NRDLD dataset pocket was calculated using the formula: (100*|#druggable—#less-druggable|)/(total number of predictions). This measure enabled us to identify how many homologous pockets gave us the same prediction regardless of the prediction itself.

### Identification of crystal structures for *P*. *aeruginosa* proteins and their homologs

The AEROPATH database (aeropath.lifesci.dundee.ac.uk, current release from 2010) contains lists of crystal structures for *P*. *aeruginosa* proteins and their homologs. These structures were downloaded from the in-house RCSB PDB mirror and subjected to structure preparation as detailed above. Only those structures were retained where at least one ligand was obtained. Repeated occurrences of the same ligand within the same PDB chain were not retained as it is rare that the same ligand occupies different pockets in the same protein structure. An exception to this rule was RmlA, where prior knowledge about a second pocket binding the cocrystallized ligand was available; hence, a separate analysis was conducted in order to include both, the active site and the allosteric pocket.

### Druggability predictions for gene products for which only crystal structure from homologous proteins were available

For each parent gene product it was ensured that the binding sites of the crystal structures of the homologous proteins covered the same sequence segment (Fig 2B). For final assessment, only corresponding pockets represented in at least five different crystal structures were retained. A protein pocket was considered to be druggable, if at least 80% consensus was reached among the homologs and the majority of the pockets were predicted druggable. For this purpose, percent consensus between different homologous pockets belonging to the same parent entry was calculated using the formula: (100*|#druggable—#less-druggable|)/(total number of predictions).

## Supporting Information

S1 File Modified NRDLD set together with descriptor values and predictions (Table A). Results from homolog-based druggability predictions for the NRDLD dataset structures when using an ambiguous zone (Table B). List of PDB 3-letter codes for cofactors or common additives during the crystallography process. These compounds were not considered to mark a binding pocket (Table C). Scripts are available on https://github.com/ruthbrenk/DrugPred2.0.git.
(DOCX)
