# Supplementary material for: To Hit or Not to Hit, That Is the Question – Genome-wide Structure-Based Druggability Predictions for Pseudomonas aeruginosa Proteins
Source: PLoS One. 2015 Sep 11;10(9):e0137279. doi: 10.1371/journal.pone.0137279 (PMC4567284; doi:10.1371/journal.pone.0137279)
Supplement: S1 File — Results from homolog-based druggability predictions for the NRDLD dataset structures when using an ambiguous zone (Table B). List of PDB 3-letter codes for cofactors or common additives during the crystallography process. These compounds were not considered to mark a binding pocket (Table C). Scripts are available on https://github.com/ruthbrenk/DrugPred2.0.git. (DOCX) [file pone.0137279.s001.docx]

**SUPPORTING INFORMATION**

To Hit or Not to Hit, That Is the Question – Genome-wide Structure-Based Druggability Predictions for *Pseudomonas aeruginosa* Proteins

Aurijit Sarkar^1^, Ruth Brenk^1,2,3^*

^1^ Division of Biological Chemistry & Drug Discovery, College of Life Sciences, University of Dundee, Dow Street, Dundee DD1 5EH, United Kingdom

^2^ Institut für Pharmazie und Biochemie, Johannes Gutenberg-Universität Mainz, , Staudinger Weg 5, 55128 Mainz, Germany

^3^ University of Bergen, Department for Biomedicine, Jonas Lies vei 91, 5020 Bergen, Norway

*Corresponding author

E-mail: : ruth.brenk@uib.no (RB)

**SUPPLEMENTARY TABLES**

**S1 Table A Modified NRDLD set together with descriptor values and predictions.**

| **Abbreviation** | **PDB code** | **ligand** | **ion** | **Co-factor** | **category** | **set** | **csa** | **hsa_t** | **psa_r** | **hiaa** | **haa** | **Prediction*** |
| --- | --- | --- | --- | --- | --- | --- | --- | --- | --- | --- | --- | --- |
| IDH | 1ai2 | ICA |  | NAP | less druggable | training | 464.00 | 207.56 | 0.55 | -1.11 | 0.11 | 0.01 |
| AAT | 1ajs | PLA |  |  | less druggable | training | 691.98 | 386.58 | 0.44 | -0.17 | 0.35 | 0.57 |
| RACE | 1b74 | DGN |  |  | less druggable | validation | 409.88 | 187.58 | 0.54 | 0.39 | 0.28 | 0.27 |
| beta-lacta | 1bls | IPP |  |  | less druggable | validation | 490.12 | 213.40 | 0.56 | -0.75 | 0.17 | 0.09 |
| ICE | 1bmq | MNO |  |  | less druggable | validation | 445.98 | 223.77 | 0.50 | -0.73 | 0.23 | 0.19 |
| ENR | 1c14 | TCL |  | NAD | druggable | training | 651.97 | 430.75 | 0.34 | 0.36 | 0.45 | 0.82 |
| OTC | 1c9y | NVA |  |  | less druggable | training | 369.95 | 168.42 | 0.54 | -1.62 | 0.17 | -0.06 |
| AdSS | 1cg0 | HDA | MG | GDP | less druggable | training | 301.39 | 161.31 | 0.46 | -0.52 | 0.19 | 0.14 |
| ATCase | 1d09 | PAL | ZN |  | less druggable | training | 598.49 | 274.59 | 0.54 | -1.48 | 0.18 | 0.10 |
| AChE | 1e66 | HUX |  |  | druggable | validation | 819.56 | 537.93 | 0.34 | 0.03 | 0.28 | 0.78 |
| CYP51 | 1e9x | PIM |  | HEM | druggable | training | 703.97 | 496.46 | 0.29 | 0.54 | 0.50 | 0.98 |
| GlucD | 1ec9 | XYH | MG |  | less druggable | validation | 210.50 | 68.47 | 0.67 | -1.93 | 0.14 | -0.37 |
| HylP2 | 1f9g | ASC |  |  | less druggable | training | 939.36 | 396.71 | 0.58 | -1.64 | 0.16 | 0.21 |
| HIV_RT_NN | 1fk9 | EFZ |  |  | druggable | validation | 693.03 | 528.19 | 0.24 | -0.08 | 0.37 | 0.90 |
| AcpS | 1fth | A3P |  |  | less druggable | training | 752.76 | 405.19 | 0.46 | -0.72 | 0.21 | 0.42 |
| KDOPS | 1g7v | PAI |  |  | less druggable | training | 892.44 | 485.54 | 0.46 | -1.29 | 0.26 | 0.48 |
| PGI | 1g98 | PA5 |  |  | less druggable | validation | 500.29 | 217.19 | 0.57 | -0.95 | 0.15 | 0.05 |
| MMP-9 | 1gkc | BUM | ZN |  | druggable | training | 512.68 | 295.45 | 0.42 | -0.37 | 0.37 | 0.47 |
| transketol | 1gpu | THD | CA |  | less druggable | training | 552.45 | 306.81 | 0.44 | -0.45 | 0.27 | 0.39 |
| HOR | 1gwr | EST |  |  | druggable | training | 564.30 | 446.28 | 0.21 | 1.79 | 0.73 | 1.30 |
| arginase | 1hqg | ORN | MN |  | less druggable | training | 514.09 | 202.53 | 0.61 | -1.89 | 0.06 | -0.16 |
| HIV_protea | 1hvr | XK2 |  |  | druggable | training | 796.81 | 507.33 | 0.36 | 0.51 | 0.45 | 0.90 |
| TS | 1hvy | D16 |  | UMP | druggable | training | 930.67 | 590.92 | 0.37 | -0.32 | 0.36 | 0.83 |
| HMG-CoA | 1hw8 | 114 |  |  | druggable | training | 624.11 | 415.36 | 0.33 | 0.04 | 0.43 | 0.76 |
| PDF | 1icj | 2PE | NI |  | less druggable | training | 756.10 | 404.47 | 0.47 | -0.28 | 0.45 | 0.62 |
| FKBP12 | 1j4i | TST |  |  | druggable | training | 405.52 | 291.86 | 0.28 | 0.84 | 0.44 | 0.78 |
| HEXB | 1jak | IFG |  |  | less druggable | training | 417.56 | 259.38 | 0.38 | -1.51 | 0.18 | 0.21 |
| DDC | 1js3 | 142 |  | PLP | druggable | training | 780.55 | 514.49 | 0.34 | -0.53 | 0.26 | 0.68 |
| TrpS | 1k7f | IAV |  |  | druggable | training | 913.70 | 616.78 | 0.32 | 0.57 | 0.47 | 1.06 |
| GL | 1k8q | C11 |  |  | druggable | training | 848.00 | 688.92 | 0.19 | 1.05 | 0.55 | 1.34 |
| PPDK | 1kc7 | PPR | MG |  | less druggable | validation | 127.13 | 43.57 | 0.66 | -1.23 | 0.17 | -0.29 |
| CDK2 | 1ke6 | LS2 |  |  | druggable | training | 778.30 | 477.88 | 0.39 | -0.42 | 0.32 | 0.66 |
| thrombin | 1kts | C24 |  |  | less druggable | training | 900.85 | 549.46 | 0.39 | -0.50 | 0.36 | 0.75 |
| PLA2 | 1kvo | OAP | CA |  | druggable | training | 1044.35 | 691.74 | 0.34 | 0.10 | 0.30 | 0.97 |
| DNA_gyrase | 1kzn | CBN |  |  | druggable | validation | 574.72 | 373.76 | 0.35 | 0.32 | 0.42 | 0.73 |
| LOX | 1lox | RS7 | FE |  | druggable | validation | 594.94 | 466.26 | 0.22 | 1.12 | 0.56 | 1.12 |
| fXa | 1lpz | CMB |  |  | druggable | training | 682.59 | 338.15 | 0.50 | -0.80 | 0.19 | 0.29 |
| DGD | 1m0n | HCP |  |  | less druggable | validation | 712.87 | 442.99 | 0.38 | 0.13 | 0.47 | 0.79 |
| EGFR | 1m17 | AQ4 |  |  | druggable | training | 814.66 | 532.35 | 0.35 | -0.15 | 0.43 | 0.85 |
| HGR | 1m2z | DEX |  |  | druggable | training | 558.38 | 385.12 | 0.31 | 1.41 | 0.61 | 1.03 |
| PLC | 1mai | I3P |  |  | less druggable | validation | 302.30 | 154.49 | 0.49 | -1.56 | 0.19 | -0.01 |
| GlmS | 1moq | GLP |  |  | less druggable | training | 509.52 | 272.80 | 0.46 | -0.01 | 0.44 | 0.50 |
| TGT | 1n2v | BDI |  |  | druggable | training | 760.47 | 407.08 | 0.46 | 0.17 | 0.32 | 0.60 |
| cathepsin | 1nlj | 2CA |  |  | less druggable | training | 450.61 | 208.97 | 0.54 | -0.50 | 0.33 | 0.23 |
| neuraminid | 1nnc | ZMR | CA |  | less druggable | training | 559.60 | 237.82 | 0.58 | -1.90 | 0.10 | -0.07 |
| ADA | 1o5r | FR9 | ZN |  | druggable | training | 955.76 | 602.99 | 0.37 | -0.42 | 0.39 | 0.85 |
| RpIA | 1o8b | ABF |  |  | less druggable | training | 403.22 | 201.02 | 0.50 | -0.74 | 0.23 | 0.16 |
| xylanase | 1od8 | XDL |  |  | less druggable | validation | 409.96 | 219.36 | 0.46 | -2.20 | 0.04 | -0.08 |
| EGase | 1olq | NAG |  |  | less druggable | validation | 97.82 | 32.18 | 0.67 | -0.25 | 0.18 | -0.19 |
| PTP1B | 1onz | 968 |  |  | less druggable | training | 516.22 | 270.72 | 0.48 | -0.74 | 0.24 | 0.27 |
| CA_II | 1oq5 | CEL | ZN |  | druggable | validation | 599.37 | 414.74 | 0.31 | 0.25 | 0.42 | 0.80 |
| JNK | 1pmn | 984 |  |  | druggable | validation | 836.18 | 505.28 | 0.40 | 0.38 | 0.43 | 0.85 |
| AR | 1pwm | FID |  | NAP | druggable | validation | 960.51 | 607.91 | 0.37 | -0.23 | 0.42 | 0.90 |
| beta-galac | 1px4 | IPT | MG |  | less druggable | validation | 856.09 | 476.53 | 0.44 | -1.22 | 0.17 | 0.43 |
| GSK | 1q41 | IXM |  |  | druggable | validation | 844.38 | 435.58 | 0.48 | -0.14 | 0.44 | 0.66 |
| TK | 1qhi | BPG |  |  | druggable | training | 538.59 | 369.55 | 0.31 | -0.74 | 0.39 | 0.61 |
| PBP-2X | 1qmf | KEF |  |  | less druggable | training | 846.10 | 462.22 | 0.45 | -0.60 | 0.33 | 0.58 |
| Lck_kinase | 1qpe | PP2 |  |  | druggable | training | 721.78 | 462.93 | 0.36 | 0.28 | 0.36 | 0.77 |
| HIV_integr | 1qs4 | 100 | MG |  | less druggable | training | 234.44 | 100.60 | 0.57 | -1.65 | 0.07 | -0.24 |
| CS | 1qxo | EPS |  | FMN | less druggable | training | 1004.81 | 502.35 | 0.50 | -1.41 | 0.14 | 0.39 |
| ADAM | 1r55 | 97 | ZN |  | druggable | validation | 551.24 | 306.04 | 0.44 | 0.19 | 0.42 | 0.56 |
| MetAP2 | 1r58 | AO5 | MN |  | druggable | training | 788.88 | 482.68 | 0.39 | -0.53 | 0.28 | 0.62 |
| CYP2E1 | 1r9o | FLP |  | HEM | druggable | training | 878.59 | 690.79 | 0.21 | 0.52 | 0.49 | 1.22 |
| ribonuclea | 1rnt | 2GP |  |  | less druggable | training | 418.97 | 192.41 | 0.54 | -2.18 | 0.11 | -0.13 |
| PNP | 1rsz | DIH |  |  | druggable | training | 820.77 | 530.21 | 0.35 | -0.10 | 0.43 | 0.85 |
| MDM2 | 1rv1 | IMZ |  |  | druggable | training | 1133.07 | 832.84 | 0.26 | 0.49 | 0.46 | 1.31 |
| HPPD | 1sqi | 869 | FE |  | druggable | training | 973.51 | 639.97 | 0.34 | 0.20 | 0.43 | 1.01 |
| HPR | 1sqn | NDR |  |  | druggable | validation | 516.60 | 340.47 | 0.34 | 0.11 | 0.46 | 0.70 |
| HIV_RT_nu | 1t03 | TFO |  |  | less druggable | training | 512.72 | 302.15 | 0.41 | -0.50 | 0.35 | 0.46 |
| c-Kit_kinase | 1t46 | STI |  |  | druggable | validation | 1037.36 | 653.81 | 0.37 | -0.06 | 0.43 | 0.98 |
| AMY | 1u30 | LAG | CA |  | druggable | training | 708.87 | 425.11 | 0.40 | -0.73 | 0.28 | 0.53 |
| ACK | 1u4d | DBQ |  |  | druggable | training | 903.82 | 524.09 | 0.42 | -0.44 | 0.33 | 0.70 |
| NDPK | 1ucn | ADP | CA |  | less druggable | training | 771.71 | 447.53 | 0.42 | -1.62 | 0.13 | 0.33 |
| PDE_5 | 1udt | VIA | ZN |  | druggable | training | 1065.61 | 705.50 | 0.34 | 0.22 | 0.40 | 1.06 |
| CDK5 | 1unl | RRC |  |  | druggable | validation | 543.46 | 289.05 | 0.47 | -0.19 | 0.41 | 0.48 |
| BCKD | 1v16 | TDP |  |  | less druggable | validation | 562.78 | 312.45 | 0.44 | -0.81 | 0.23 | 0.33 |
| HK | 1v4s | MRK |  |  | druggable | training | 872.14 | 536.33 | 0.39 | -0.27 | 0.37 | 0.78 |
| TyrRS | 1vbm | YSA |  |  | druggable | training | 1041.61 | 557.57 | 0.46 | -0.29 | 0.40 | 0.77 |
| CDP-D-gluc | 1wvc | CTP | MG |  | less druggable | validation | 839.11 | 457.18 | 0.46 | -0.19 | 0.41 | 0.36 |
| DPP-IV | 1X70 | 715 |  |  | druggable | training | 935.15 | 521.60 | 0.44 | -1.02 | 0.13 | 0.47 |
| Mandase | 1x9d | SMD | CA |  | less druggable | training | 525.92 | 254.38 | 0.52 | -1.24 | 0.30 | 0.20 |
| PDE_4B | 1xm6 | 5RM | MG |  | druggable | training | 871.23 | 519.72 | 0.40 | -0.44 | 0.29 | 0.67 |
| PDE_5A | 1xoz | CIA | MG |  | druggable | validation | 940.24 | 673.03 | 0.28 | -0.20 | 0.43 | 0.68 |
| LF | 1yqy | 915 | ZN |  | druggable | training | 1047.88 | 605.50 | 0.42 | -1.05 | 0.20 | 0.63 |
| HCV_NS5B | 1yvf | PH7 |  |  | druggable | training | 945.10 | 567.22 | 0.40 | -0.16 | 0.40 | 0.83 |
| VEGFR2 | 1ywn | LIF |  |  | druggable | training | 1005.99 | 686.79 | 0.32 | 0.77 | 0.41 | 1.13 |
| p38_kinase | 1ywr | LI9 |  |  | druggable | training | 773.40 | 509.93 | 0.34 | 0.73 | 0.49 | 0.97 |
| HMR | 2aa2 | AS4 |  |  | druggable | validation | 496.37 | 316.87 | 0.36 | 1.67 | 0.65 | 1.03 |
| CHK1 | 2br1 | PFP |  |  | druggable | training | 646.80 | 408.58 | 0.37 | -0.40 | 0.41 | 0.66 |
| MAO_A | 2bxr | MLG |  | FAD | druggable | training | 788.24 | 491.25 | 0.38 | 0.74 | 0.39 | 0.86 |
| COMT | 2cl5 | BIE | MG | SAM | druggable | validation | 1153.16 | 771.77 | 0.33 | -0.16 | 0.41 | 0.97 |
| Fyn_kinase | 2dq7 | STU |  |  | druggable | training | 916.13 | 571.24 | 0.38 | 0.07 | 0.33 | 0.83 |
| BRAF | 2fb8 | 215 |  |  | druggable | training | 950.71 | 602.99 | 0.37 | -0.13 | 0.42 | 0.91 |
| renin | 2g24 | 7IG |  |  | druggable | training | 995.80 | 653.39 | 0.34 | 0.30 | 0.40 | 1.01 |
| GR | 2gh5 | ELI |  | FAD | druggable | training | 707.27 | 410.47 | 0.42 | -0.34 | 0.39 | 0.62 |
| NPP | 2gsu | AMP | ZN |  | less druggable | training | 896.28 | 520.82 | 0.42 | -1.46 | 0.14 | 0.44 |
| XI | 2gyi | HYA | MG |  | less druggable | training | 218.78 | 135.90 | 0.38 | -1.22 | 0.18 | 0.11 |
| Abl_kinase | 2hiw | 7MP |  |  | druggable | training | 1262.59 | 815.95 | 0.35 | 0.06 | 0.36 | 1.12 |
| PCK | 2i0e | PDS |  |  | druggable | training | 640.92 | 400.73 | 0.37 | -0.12 | 0.34 | 0.64 |
| FMS_kinase | 2i1m | 5CN |  |  | druggable | validation | 922.58 | 525.32 | 0.43 | -0.54 | 0.26 | 1.10 |
| RET | 2ivu | ZD6 |  |  | druggable | training | 945.16 | 590.38 | 0.38 | -0.25 | 0.42 | 0.87 |
| HAR | 3b68 | B68 |  |  | druggable | validation | 691.64 | 518.04 | 0.25 | 0.02 | 0.47 | 0.96 |
| GPCR | 3d4s | TIM |  |  | druggable | training | 793.63 | 529.43 | 0.33 | 0.30 | 0.38 | 0.87 |
| XOR | 3etr | LUZ |  | MTE | druggable | validation | 535.04 | 380.29 | 0.29 | 0.06 | 0.38 | 0.73 |
| HDAC_8 | 3f0r | TSN | ZN |  | druggable | validation | 775.29 | 535.81 | 0.31 | -0.15 | 0.32 | 0.81 |
| DHODH | 3f1q | BCE |  | FMN | druggable | validation | 834.20 | 647.39 | 0.22 | 0.94 | 0.58 | 1.28 |
| DHFR | 3ia4 | MTX |  | NDP | druggable | validation | 633.15 | 355.89 | 0.44 | -0.15 | 0.44 | 0.60 |
| AGAT | 3jdw | ORN |  |  | less druggable | validation | 407.36 | 229.44 | 0.44 | -0.98 | 0.24 | 0.23 |
| PCD | 3pcm | NNO | FE |  | less druggable | training | 738.66 | 451.72 | 0.39 | -1.46 | 0.16 | 0.40 |
| COX_2 | 4cox | IMN |  | HEM | druggable | training | 802.88 | 598.50 | 0.25 | 1.00 | 0.55 | 1.20 |
| * Binding sites with values ≤ 0.50 were considered to be less druggable and with > 0.50 druggable. If an ambiguous zone was applied, binding sites with values from 0.4-0.6 were disregarded. | | | | | | | | | | | | |

S1 Table B Results from homolog-based druggability predictions for the NRDLD dataset structures when using an ambiguous zone.

| **NRDLD dataset structure** | **Minimum % sequence ID** | **Maximum % sequence ID** | **# homologous pockets studied** | **# predicted druggable** | **# predicted less druggable** | **Parent Protein druggable?** | **Minimum % sequence ID for 100% correct predictions** | **% correct predictions** |
| --- | --- | --- | --- | --- | --- | --- | --- | --- |
| 1ai2 | 24. 9 | 32.3 | 2 | 0 | 2 | FALSE | 24.9 | 100 |
| 1b74 | 42.4 | 47.1 | 6 | 0 | 6 | FALSE | 42.4 | 100 |
| 1bls | 45.6 | 76.2 | 34 | 4 | 30 | FALSE | 72 | 88.2 |
| 1bmq | 34.3 | 34.3 | 15 | 0 | 15 | FALSE | 34.3 | 100 |
| 1c9y | 22.6 | 37.5 | 24 | 2 | 22 | FALSE | 22 | 91. 7 |
| 1d09 | 28.3 | 54.2 | 5 | 0 | 5 | FALSE | 28.3 | 100 |
| 1e66 | 33.4 | 60.7 | 22 | 19 | 3 | TRUE | 60 | 86. 4 |
| 1ec9 | 23.4 | 65.6 | 6 | 0 | 6 | FALSE | 23.4 | 100 |
| 1f9g | 24.4 | 24.4 | 1 | 0 | 1 | FALSE | 24.4 | 100 |
| 1g7v | 49.8 | 69.2 | 3 | 0 | 3 | FALSE | 49.8 | 100 |
| 1g98 | 45.3 | 64.0 | 5 | 0 | 5 | FALSE | 45.3 | 100 |
| 1gkc | 54.6 | 64.7 | 28 | 25 | 3 | TRUE | 55 | 89.3 |
| 1gpu | 26.4 | 47.5 | 3 | 0 | 3 | FALSE | 26.4 | 100 |
| 1hqg | 35.7 | 65.8 | 5 | 0 | 5 | FALSE | 35.7 | 100 |
| 1hvr | 86.8 | 89.5 | 7 | 7 | 0 | TRUE | 86.8 | 100 |
| 1hvy | 44.2 | 44.2 | 1 | 0 | 1 | TRUE | - | 0 |
| 1icj | 32.5 | 58.4 | 18 | 15 | 3 | FALSE | 58 | 16. 7 |
| 1j4i | 44.6 | 61.6 | 4 | 4 | 0 | TRUE | 44.6 | 100 |
| 1jak | 24.2 | 52.6 | 7 | 2 | 5 | FALSE | 26 | 71.4 |
| 1kc7 | 56.8 | 56.8 | 1 | 0 | 1 | FALSE | 56.8 | 100 |
| 1ke6 | 32.6 | 63.7 | 40 | 40 | 0 | TRUE | 32.6 | 100 |
| 1kts | 36.5 | 84.8 | 162 | 34 | 128 | FALSE | 84 | 79 |
| 1kvo | 38.2 | 53 | 37 | 37 | 0 | TRUE | 38.2 | 100 |
| 1kzn | 44 | 84.7 | 7 | 7 | 0 | TRUE | 44 | 100 |
| 1lox | 27.5 | 27.5 | 1 | 1 | 0 | TRUE | 27.5 | 100 |
| 1lpz | 35.6 | 61 | 148 | 36 | 112 | TRUE | 61 | 24.3 |
| 1m0n | 24.7 | 34 | 14 | 11 | 3 | FALSE | 33 | 21.4 |
| 1m17 | 25.9 | 82.6 | 96 | 96 | 0 | TRUE | 25.9 | 100 |
| 1moq | 31.5 | 31.5 | 1 | 0 | 1 | FALSE | 31.5 | 100 |
| 1n2v | 30.0 | 30.0 | 2 | 2 | 0 | TRUE | 30.0 | 100 |
| 1nlj | 25.2 | 59.1 | 58 | 9 | 49 | FALSE | 58 | 84.5 |
| 1nnc | 33.2 | 69.1 | 34 | 3 | 31 | FALSE | 49 | 91.2 |
| 1o5r | 27.9 | 84.5 | 7 | 7 | 0 | TRUE | 27.9 | 100 |
| 1o8b | 50.9 | 80 | 6 | 1 | 5 | FALSE | 60 | 83.3 |
| 1od8 | 28.6 | 89.7 | 21 | 2 | 19 | FALSE | 49 | 90.5 |
| 1onz | 36.6 | 42.2 | 7 | 0 | 7 | FALSE | 36.6 | 100 |
| 1oq5 | 36.1 | 62.5 | 9 | 8 | 1 | TRUE | 36 | 88. 9 |
| 1owe | 28.3 | 45.0 | 208 | 68 | 140 | TRUE | 41 | 32.7 |
| 1pmn | 23.4 | 56.9 | 102 | 102 | 0 | TRUE | 23.4 | 100 |
| 1pwm | 48 | 87.5 | 29 | 29 | 0 | TRUE | 48.0 | 100 |
| 1px4 | 28 | 30.0 | 2 | 1 | 1 | FALSE | 28.0 | 50 |
| 1q41 | 28.6 | 35.4 | 103 | 103 | 0 | TRUE | 28.6 | 100 |
| 1qhi | 39.5 | 39.5 | 3 | 1 | 2 | TRUE | 39 | 33.3 |
| 1qpe | 23.2 | 74.2 | 85 | 85 | 0 | TRUE | 23.2 | 100 |
| 1r55 | 37.9 | 41.1 | 3 | 1 | 2 | TRUE | 41 | 33.3 |
| 1r58 | 47.4 | 47.4 | 2 | 2 | 0 | TRUE | 47.4 | 100 |
| 1r9o | 22.2 | 78.3 | 14 | 14 | 0 | TRUE | 22.2 | 100 |
| 1rnt | 68.7 | 73.0 | 3 | 0 | 3 | FALSE | 68.7 | 100 |
| 1rsz | 25.8 | 89.9 | 29 | 21 | 8 | TRUE | 89 | 72.4 |
| 1x70 | 26.8 | 88.9 | 12 | 0 | 12 | TRUE | - | 0 |
| 1sqi | 48.1 | 48.1 | 1 | 1 | 0 | TRUE | 48.1 | 100 |
| 1t46 | 23.4 | 56.9 | 94 | 94 | 0 | TRUE | 23.4 | 100 |
| 1u30 | 22.4 | 86.1 | 4 | 0 | 4 | TRUE | - | 0 |
| 1u4d | 28.5 | 42.4 | 97 | 97 | 0 | TRUE | 28.5 | 100 |
| 1ucn | 38.6 | 86.5 | 40 | 12 | 28 | FALSE | 61 | 70 |
| 1udt | 23.9 | 43.4 | 28 | 28 | 0 | TRUE | 23.9 | 100 |
| 1unl | 30.9 | 65 | 123 | 123 | 0 | TRUE | 30.9 | 100 |
| 1v16 | 29.7 | 35.5 | 8 | 7 | 1 | FALSE | 35 | 12.5 |
| 1vbm | 31.5 | 52.7 | 6 | 5 | 1 | TRUE | 42 | 83.3 |
| 1xm6 | 28.1 | 86.1 | 28 | 28 | 0 | TRUE | 28.1 | 100 |
| 1xoz | 28 | 42.3 | 31 | 31 | 0 | TRUE | 28 | 100 |
| 1yvf | 89 | 89 | 1 | 1 | 0 | TRUE | 89.0 | 100 |
| 1ywn | 27.4 | 89.7 | 83 | 83 | 0 | TRUE | 27.4 | 100 |
| 1ywr | 27.1 | 58.4 | 107 | 107 | 0 | TRUE | 27.1 | 100 |
| 2br1 | 27.9 | 40.4 | 97 | 95 | 2 | TRUE | 34 | 97.9 |
| 2bxr | 73.3 | 73.3 | 7 | 7 | 0 | TRUE | 73.3 | 100 |
| 2dq7 | 24.8 | 80.3 | 105 | 105 | 0 | TRUE | 24.8 | 100 |
| 2fb8 | 23.7 | 36.4 | 116 | 116 | 0 | TRUE | 23.7 | 100 |
| 2g24 | 24.6 | 41.1 | 93 | 52 | 41 | TRUE | 32 | 55.9 |
| 2gh5 | 34.3 | 36.9 | 2 | 2 | 0 | TRUE | 34.3 | 100 |
| 2gsu | 29.2 | 29.2 | 1 | 1 | 0 | FALSE | - | 0 |
| 2gyi | 26.1 | 68.1 | 20 | 1 | 19 | FALSE | 63 | 95 |
| 2hiw | 28.3 | 51.5 | 98 | 98 | 0 | TRUE | 28.3 | 100 |
| 2i1m | 28.7 | 58.5 | 105 | 105 | 0 | TRUE | 28.7 | 100 |
| 2ivu | 26.2 | 48.4 | 128 | 128 | 0 | TRUE | 26.2 | 100 |
| 3etr | 87.6 | 87.6 | 1 | 1 | 0 | TRUE | 87.6 | 100 |
| 3f0r | 29.4 | 29.4 | 1 | 1 | 0 | TRUE | 29.4 | 100 |
| 3f1q | 40.2 | 89.3 | 5 | 5 | 0 | TRUE | 40.2 | 100 |
| 3ia4 | 30.2 | 59.5 | 168 | 168 | 0 | TRUE | 30.2 | 100 |
| 4cox | 65.8 | 65.8 | 5 | 5 | 0 | TRUE | 65.8 | 100 |
| 1ajs | 22.3 | 81.8 | 43 | 19 | 24 | FALSE | 47 | 55.8 |
| 2i0e | 26.7 | 46 | 98 | 95 | 3 | TRUE | 39 | 96.9 |

S1 Table C List of PDB codes for cofactors or common additives during the crystallography process. These compounds were not considered to mark a binding pocket.

| 1FH | 12-PHENYLHEME |
| --- | --- |
| 202 | BROMIC ACID |
| 2BM | DIBROMOMETHANE |
| 2FH | 2-PHENYLHEME |
| 2FU | BUT-2-ENEDIAL |
| 2ME | METHOXYETHANE |
| 2MO | MOLYBDENUM (IV)OXIDE |
| 2NO | NITROGEN DIOXIDE |
| 2OF | FERROUS ION, 2 WATERS COORDINATED |
| 2PA | DIAMIDOPHOSPHATE |
| 2PN | IMIDODIPHOSPHORIC ACID |
| 2PO | PHOSPHONATE |
| 3BR | 1-BROMOPROPANE |
| 3CN | 3-AMINOPROPANE |
| 3CO | COBALT (III) ION |
| 3OF | HYDRATED FE (III) ION, 2 WATERS COORDINATED |
| 543 | CALCIUM ION, 6 WATERS PLUS ETHANOL COORDINATED |
| 6WO | OXO-TUNGSTEN(VI) |
| ACT | ACETATE ION |
| ACU | ACETALDEHYDE |
| AEM | 2-AMINOETHANIMIDIC ACID |
| AF3 | ALUMINUM FLUORIDE |
| AL | ALUMINUM ION |
| AR | ARGON |
| ARS | ARSENIC |
| ATO | CHLOROACETONE |
| AU | GOLD ION |
| AU3 | GOLD 3+ ION |
| AUC | GOLD (I) CYANIDE ION |
| AZI | AZIDE ION |
| BBU | 1-BROMOBUTANE |
| BBX | 1-BROMOETHANE |
| BEF | BERYLLIUM TRIFLUORIDE ION |
| BF2 | BERYLLIUM DIFLUORIDE |
| BF4 | BERYLLIUM TETRAFLUORIDE ION |
| BO4 | BORATE ION |
| BRJ | 2-BROMOETHANOL |
| BRM | 2-BROMOACETYL GROUP |
| BRP | 2-BROMO-2-PROPENE-1-OL |
| C10 | HEXAETHYLENE GLYCOL MONODECYL ETHER |
| C2C | CU-CL-CU LINKAGE |
| C2O | CU-O-CU LINKAGE |
| CAC | CACODYLATE ION |
| CB5 | COBALT BIS(1,2-DICARBOLLIDE) |
| CCH | [7-ETHENYL-12-FORMYL-3,8,13,17-TERTRAMETHYL-21H,23H-PORPHINE-2,18-DIPROPANOATO(2)-N21,N22,N23,N24]IRON |
| CCN | ACETONITRILE |
| CD1 | CADMIUM ION, 1 WATER COORDINATED |
| CD3 | CADMIUM ION, 3 WATERS COORDINATED |
| CD5 | CADMIUM ION, 5 WATERS COORDINATED |
| CE | CERIUM (III) ION |
| CEQ | ETHYL-TRIMETHYL-SILANE |
| CFM | FE-MO-S CLUSTER |
| CFN | FE(7)-MO-S(9)-N CLUSTER |
| CFO | CHLORO DIIRON-OXO MOIETY |
| CFT | TRIFLUOROMETHANE |
| CHM | 1,3-DICHLORO-PROPAN-2-ONE |
| CLF | FE(8)-S(7) CLUSTER |
| CLN | SULFUR SUBSTITUTED PROTOPORPHYRIN IX |
| CLP | FE-S CLUSTER |
| CN1 | OXO-IRON CLUSTER 2 |
| CNB | OXO-IRON CLUSTER 1 |
| CNF | OXO-IRON CLUSTER 3 |
| CNM | ACETAMIDE |
| CO5 | COBALT ION,5 WATERS COORDINATED |
| COH | PROTOPORPHYRIN IX CONTAINING CO |
| CON | COBALT TETRAAMMINE ION |
| CP2 | 1,2-DICHLORO-PROPANE |
| CPT | CIS-PLATINUM-(NH3)2 |
| CR | CHROMIUM ION |
| CS | CESIUM ION |
| CUA | DINUCLEAR COPPER ION |
| CUL | COPPER (II) CHLORIDE |
| CUM | CU(I)-S-MO(VI)(=O)OH CLUSTER |
| CUN | CU(I)-S-MO(IV)(=O)OH CLUSTER |
| CUO | CU2-O2 CLUSTER |
| CUZ | (MU-4-SULFIDO)-TETRA-NUCLEAR COPPER ION |
| DDH | [7,12-DEACETYL-3,8,13,17-TETRAMETHYL-21H,23H-PORPHINE-2,18-DIPROPANOATO(2-)-N21,N22,N23,N24]-IRON |
| DHE | HEME D |
| DIS | DISORDERED SOLVENT |
| DOD | DEUTERATED WATER |
| DPO | DIPHOSPHATE |
| DTN | DITHIONITE |
| DUM | DUMMY ATOMS |
| DVT | DECAVANADATE |
| DXE | 1,2-DIMETHOXYETHANE |
| DZZ | O,O-DIMETHYL HYDROGEN THIOPHOSPHATE |
| E1H | ETHANIMINE |
| EGL | ETHYLENE GLYCOL |
| EHN | ETHANE |
| EMC | ETHYL MERCURY ION |
| ENC | ETHYL ISOCYANIDE |
| EOM | ETHYLOXYMETHOXYL |
| ETF | TRIFLUOROETHANOL |
| ETI | IODOETHANE |
| ETN | METHYLETHYLAMINE |
| EU | EUROPIUM ION |
| EU3 | EUROPIUM (III) ION |
| F2O | MU-OXO-DIIRON(III) |
| F3S | FE3-S4 CLUSTER |
| F50 | ETHANEPEROXOIC ACID |
| FCO | CARBONMONOXIDE-(DICYANO) IRON |
| FDD | FE(III) 2,4-DIMETHYL DEUTEROPORPHYRIN IX |
| FDE | FE(III) DEUTEROPORPHYRIN IX |
| FEA | MONOAZIDO-MU-OXO-DIIRON |
| FEC | 1,3,5,8-TETRAMETHYL-PORPHINE-2,4,6,7-TETRAPROPIONIC ACID FERROUS COMPLEX |
| FEL | HYDRATED FE |
| FEO | MU-OXO-DIIRON |
| FES | FE2/S2 (INORGANIC) CLUSTER |
| FLH | FORMALDEHYDE |
| FMI | FE-(4-MESOPORPHYRINONE)-R-ISOMER |
| FMS | TRIFLUOROMETHANE SULFONAMIDE |
| FNE | (MU-SULPHIDO)-BIS(MU-CYS,S)-[TRICARBONYLIRON-DI-(CYS,S)NICKEL(II)](FE-NI) |
| FPO | FLUORO-PHOSPHITE ION |
| FS1 | IRON/SULFUR CLUSTER |
| FS2 | FE-S-O HYBRID CLUSTER |
| FS3 | FE3-S4 CLUSTER |
| FS4 | IRON/SULFUR CLUSTER |
| FSO | IRON/SULFUR/OXYGEN HYBRID CLUSTER |
| FSX | BIS-(MU-2-OXO),[(MU-3--SULFIDO)-BIS(MU-2--SULFIDO)-TRIS(CYS-S)-TRI-IRON] (AQUA)(GLU-O)IRON(II) |
| GA | GALLIUM (III) ION |
| GAI | GUANIDINE |
| GD | GADOLINIUM ATOM |
| GD3 | GADOLINIUM ION |
| HDD | CIS-HEME D HYDROXYCHLORIN GAMMA-SPIROLACTONE |
| HDN | METHYLHYDRAZINE |
| HE5 | ZINC(II)-DEUTEROPORPHYRIN DIMETHYLESTER |
| HE6 | 6,7-DICARBOXYL-1,2,3,4,5,8-HEXAMETHYLHEMIN |
| HEA | HEME-A |
| HEB | HEME B/C |
| HEC | HEME C |
| HES | ZINC SUBSTITUTED HEME C |
| HEV | 5,8-DIMETHYL-1,2,3,4-TETRAVINYLPORPHINE-6,7-DIPROPIONIC ACID FERROUS COMPLEX |
| HFM | 2-FORMYL-PROTOPORPHRYN IX |
| HG2 | DIBROMOMERCURY |
| HGC | METHYL MERCURY ION |
| HGI | MERCURY (II) IODIDE |
| HLT | 2-BROMO-2-CHLORO-1,1,1-TRIFLUOROETHANE |
| HNI | PROTOPORPHYRIN IX CONTAINING NI(II) |
| HO | HOLMIUM ATOM |
| HO3 | HOLMIUM (III) ATOM |
| HSW | N-hydroxysulfamide |
| I42 | HYDROXY(DIOXIDO)OXOVANADIUM |
| IN | INDIUM (III) ION |
| IOH | 2-PROPANOL, ISOPROPANOL |
| IR | IRIDIUM ION |
| IR3 | IRIDIUM (III) ION |
| IRI | IRIDIUM HEXAMMINE ION |
| IUM | URANYL (VI) ION |
| KO4 | POTASSIUM ION, 4 WATERS COORDINATED |
| KR | KRYPTON |
| LA | LANTHANUM (III) ION |
| LCP | PERCHLORATE ION |
| LI | LITHIUM ION |
| LU | LUTETIUM (III) ION |
| MAC | MERCURY ACETATE ION |
| MDD | MALONALDEHYDE |
| MGF | TRIFLUOROMAGNESATE |
| MH2 | MANGANESE ION, 1 HYDROXYL COORDINATED |
| MH3 | MANGANESE ION, 1 HYDROXYL COORDINATED |
| MHM | [7,12-DIETHYL-3,8,13,17-TETRAMETHYL-21H,23H-PORPHINE-2,18-DIPORPANOTO-(2)-N21,N22,N23,N24,]IRON |
| MLP | 1-AMINOCYCLOPROPYLPHOSPHONATE |
| MM4 | MOLYBDENUM (IV) OXIDE |
| MMC | METHYL MERCURY ION |
| MN5 | MANGANESE ION, 5 WATERS COORDINATED |
| MN6 | MANGANESE ION, 6 WATERS COORDINATED |
| MNC | METHYL ISOCYANIDE |
| MNH | MANGANESE PROTOPORPHYRIN IX |
| MNR | PROTOPORPHYRIN IX CONTAINING MN |
| MO1 | MAGNESIUM ION, 1 WATER COORDINATED |
| MO3 | MAGNESIUM ION, 3 WATERS COORDINATED |
| MO4 | MAGNESIUM ION, 4 WATERS COORDINATED |
| MO5 | MAGNESIUM ION, 5 WATERS COORDINATED |
| MO6 | MAGNESIUM ION, 6 WATERS COORDINATED |
| MO7 | HEPTAMOLYBDATE |
| MOO | MOLYBDATE ION |
| MOS | DIOXOTHIOMOLYBDENUM(VI) ION |
| MP1 | N-METHYLMESOPORPHYRIN CONTAINING COPPER |
| MPR | 2-MERCAPTO-PROPION ALDEHYDE |
| MPS | METHYLPHOSPHINIC ACID |
| MSF | [METHYLSELENO]ACETATE |
| MTD | [METHYLTELLURO]ACETATE |
| MTF | N-METHYLTHIOFORMAMIDE |
| MTO | BOUND WATER |
| MW1 | MANGANESE ION, 1 WATER COORDINATED |
| MW2 | MANGANESE DIHYDRATE ION |
| MW3 | MANGANESE ION, 3 WATERS COORDINATED |
| MXE | 2-METHOXYETHANOL |
| MYQ | (S)-(1,2-DICARBOXYETHYLTHIO)GOLD |
| NA2 | SODIUM ION, 2 WATER COORDINATED |
| NA5 | SODIUM ION, 5 WATERS COORDINATED |
| NA6 | SODIUM ION, 6 WATERS COORDINATED |
| NAW | SODIUM ION, 3 WATERS COORDINATED |
| NCO | COBALT HEXAMMINE ION |
| NCP | CIS-DIAMINODICHLOROPLATINUM |
| ND4 | AMMONIUM CATION WITH D |
| NFB | NI-FE ACTIVE CENTER B-FORM |
| NFC | NI-FE ACTIVE CENTER A-FORM |
| NFE | NI-FE ACTIVE CENTER |
| NFO | NI-FE OXIDIZED ACTIVE CENTER |
| NFR | NI-FE REDUCED ACTIVE CENTER |
| NFS | FE(4)-NI(1)-S(5) CLUSTER |
| NGN | NITROGEN |
| CXE | PENTAETHYLENE GLYCOL MONODECYL ETHER |
| NI1 | NICKEL ION, 1 WATER COORDINATED |
| NI2 | NICKEL (II) ION, 2 WATERS COORDINATED |
| NI3 | NICKEL (II) ION, 3 WATERS COORDINATED |
| NIK | PENTAAQUANICKEL |
| NML | N-METHYLACETAMIDE |
| NMO | NITROGEN MONOXIDE |
| NO | NITRIC OXIDE |
| MA4 | CYCLOHEXYL-HEXYL-BETA-D-MALTOSIDE |
| NO3 | NITRATE ION |
| NOE | NITROSOETHANE |
| NRU | RUTHENIUM (III) HEXAAMINE ION |
| O | OXYGEN ATOM |
| O2 | OXYGEN MOLECULE |
| O4M | MANGANESE TETRAHYDRATE ION |
| OC1 | CALCIUM ION, 1 WATER COORDINATED |
| TLA | L(+)-TARTARIC ACID |
| OC3 | CALCIUM ION, 3 WATERS COORDINATED |
| OC4 | CALCIUM ION, 4 WATERS COORDINATED |
| OC5 | CALCIUM ION, 5 WATERS COORDINATED |
| OC6 | CALCIUM ION, 6 WATERS COORDINATED |
| OC7 | CALCIUM ION, 7 WATERS COORDINATED |
| OC8 | CALCIUM ION, 8 WATERS COORDINATED |
| OCL | COBALT ION, 1 WATER COORDINATED |
| OCM | COBALT ION, 3 WATERS COORDINATED |
| OCN | COBALT ION, 2 WATERS COORDINATED |
| OCO | COBALT ION,6 WATERS COORDINATED |
| OEC | OXYGEN EVOLVING SYSTEM |
| OF0 |  |
| OF1 | FERROUS ION, 1 WATER COORDINATED |
| OF2 | 2 FERRIC ION, 1 BRIDGING OXYGEN |
| OF3 | FERRIC ION, 1 WATER COORDINATED |
| CIT | CITRIC ACID |
| OMO | MO(VI)(=O)(OH)2 CLUSTER |
| OS | OSMIUM ION |
| OSM | 1-(OXIDOSULFANYL)METHANAMINE |
| DPG | DPHPG |
| OX | BOUND OXYGEN |
| PE3 | POLYETHYLENE GLYCOL |
| PBM | TRIMETHYL LEAD ION |
| PC3 | COPROPORPHYRIN I CONTAINING CO(III) |
| PC4 | TETRACHLOROPLATINATE(II) |
| PCL | PLATINUM(II) DI-CHLORIDE |
| PD | PALLADIUM ION |
| PDT | 1,3-PROPANEDITHIOL |
| PEJ | PERIODATE |
| B3P | BIS-TRIS PROPANE |
| PGL | AMINOMETHYLENEPHOSPHINIC ACID |
| PGQ | S-1,2-PROPANEDIOL |
| COM | 1-THIOETHANESULFONIC ACID |
| F09 | NONAN-1-OL |
| PIS | TRIHYDROGEN THIODIPHOSPHATE |
| PNL | PROPANAL |
| PO2 | HYPOPHOSPHITE |
| PO3 | PHOSPHITE ION |
| TRS | TRIS BUFFER |
| SO4 | SULFATE |
| PON | IMIDO DIPHOSPHATE |
| POP | PYROPHOSPHATE 2- |
| POR | PORPHYRIN FE(III) |
| PP9 | PROTOPORPHYRIN IX |
| GOL | GLYCEROL |
| PEG | Polyethylene glycol |
| PPK | (DIPHOSPHONO)AMINOPHOSPHONIC ACID |
| PPM | PHOSPHONOMETHYL GROUP |
| PR | PRASEODYMIUM ION |
| PS5 | PENTASULFIDE-SULFUR |
| PSL | PYROSULFATE |
| PT | PLATINUM (II) ION |
| PT4 | PLATINUM (IV) ION |
| PTL | PENTANAL |
| PTN | PLATINUM TRIAMINE ION |
| RE | RHENIUM |
| REO | PERRHENATE |
| RGI | METHYLCARBAMIC ACID |
| RHD | RHODIUM HEXAMINE ION |
| ROP | PROPIONAMIDE |
| RTC | RHENIUM (I) TRICARBONYL |
| RU | RUTHENIUM ION |
| RU7 | PARA-CYMENE RUTHENIUM CHLORIDE |
| S | SULFUR ATOM |
| S0H | (S)-2-HYDROXYPROPYLPHOSPHONIC ACID |
| SB | ANTIMONY (III) ION |
| SBE | 1,3,2-DIOXABOROLAN-2-OL |
| SBO | TRIHYDROXYANTIMONITE(III) |
| SBT | 2-BUTANOL |
| MPO | 3[N-MORPHOLINO]PROPANE SULFONIC ACID |
| SE | SELENIUM ATOM |
| PG0 | PEG 6000 |
| SEA | THIOETHYLAMINE |
| SEK | SELENOCYANATE ION |
| SF3 | FE4-S3 CLUSTER |
| SF4 | IRON/SULFUR CLUSTER |
| SFN | SULFONATE GROUP |
| SFO | SULFONIC ACID |
| SGM | MONOTHIOGLYCEROL |
| SM | SAMARIUM (III) ION |
| SMO | DIOXOSULFIDOMOLYBDENUM(VI) ION |
| PEU | PEG 8000 |
| SOM | METHYL PHOSPHINIC ACID |
| SRM | SIROHEME |
| SUL | SULFATE ANION |
| SX | SULFUR OXIDE |
| T1A | TETRAETHYLARSONIUM ION |
| TAS | TRIHYDROXYARSENITE(III) |
| TB | TERBIUM(III) ION |
| TBR | HEXATANTALUM DODECABROMIDE |
| TBU | TERTIARY-BUTYL ALCOHOL |
| TCN | TETRACYANONICKELATE ION |
| TE | TELLURIUM |
| TEE | 2-AMINO-ETHENETHIOL |
| TFA | TRIFLUOROACETYL GROUP |
| TFH | NITROGEN OF TRIFLUORO-ETHYLHYDRAZINE |
| TL | THALLIUM (I) ION |
| TSD | 3-TRIMETHYLSILYL-PROPIONATE-2,2,3,3,-D4 |
| TSE | THIOPHOSPHONOACETIC ACID |
| TSM | 2-HYDROSULFONYL-2-METHYLPROPANE |
| TZZ | TRIMETHYL PHOSPHATE |
| U1 | URANIUM ATOM |
| UNX | UNKNOWN ATOM OR ION |
| PE5 | 2-(2-{2-[2-(2-{2-[2-(2-ETHOXY-ETHOXY)-ETHOXY]-ETHOXY}-ETHOXY)-ETHOXY]-ETHOXY}-ETHOXY)-ETHANOL, POLYETHYLENE GLYCOL PEG400 |
| V | VANADIUM ION |
| V40 |  |
| V70 |  |
| VA3 | TRIVANADATE |
| VER | IRON-OCTAETHYLPORPHYRIN |
| VO3 | TETRAMETAVANADATE |
| VSO | VINYLSULPHONIC ACID |
| VX | O-ETHYLMETHYLPHOSPHONIC ACID ESTER GROUP |
| VXA | METHYLPHOSPHONIC ACID ESTER GROUP |
| WCC | FE(3)-NI(1)-S(4) CLUSTER |
| WO2 | OCTADECATUNGSTENYL DIPHOSPHATE |
| WO4 | TUNGSTATE(VI)ION |
| WO5 | TUNGSTATE(VI) ION |
| XCC | FE(4)-NI(1)-S(4) CLUSTER |
| XL1 | ETHANETHIOL |
| Y1 | YTTRIUM ION |
| YB | YTTERBIUM (III) ION |
| YBT | BIS-(2-HYDROXYETHYL)AMINO-TRIS(HYDROXYMETHYL)METHANE YTTRIUM |
| YH | YTTRIUM ION, 1 WATER COORDINATED |
| YT3 | YTTRIUM (III) ION |
| ZEM | 20-OXO-PROTOPORPHYRIN IX CONTAINING ZN(II) |
| ZH3 | ZINC TRIHYDROXIDE |
| P33 | HEPTAETHYLENE GLYCOL, PEG330 |
| ZN1 |  |
| ZN2 | ZINC ION ON 3-FOLD CRYSTAL AXIS |
| ZN3 | ZINC ION, 1 WATER COORDINATED |
| P2K | POLYETHYLENE GLYCOL PEG2000 |
| ZNO | ZINC ION, 2 WATERS COORDINATED |
| ZO3 | ZINC ION, 3 WATERS COORDINATED |
| ZRC | OXO ZIRCONIUM(IV) CLUSTER |
| BRO | BROMIDE ION |
| CBM | ACETIC ACID |
| CBX | FORMIC ACID |
| CLO | CHLORIDE ION |
| CM | ACETIC ACID |
| 15P | POLYETHYLENE GLYCOL PEG1500 |
| MPD | (4S)-2-METHYL-2,4-PENTANEDIOL |
| PG4 | TETRAETHYLENE GLYCOL |
| HTO | HEPTANE-1,2,3-TRIOL |
| MRD | (4R)-2-methylpentane-2,4-diol |
| BTB | BIS-TRIS BUFFER |
| 1PE | PEG400 |
| LDA | LAURYL DIMETHYLAMINE-N-OXIDE |
| PGE | TRIETHYLENE GLYCOL |
| 7PE | POLYETHYLENE GLYCOL FRAGMENT |
| MQD | 2-METHYLPENTANE-1,2,4-TRIOL |
| EPE | HEPES |
| 12P | POLYETHYLENE GLYCOL PEG400 |
| PG5 | Triethylene glycol dimethyl ether |
| C8E | (HYDROXYETHYLOXY)TRI(ETHYLOXY)OCTANE |
| T3A | N-(TRIS(HYDROXYMETHYL)METHYL)-3-AMINOPROPANESULFONIC ACID |
| MES | 2-(N-MORPHOLINO)-ETHANESULFONIC ACID |
| DTV | (2S,3S)-1,4-DIMERCAPTOBUTANE-2,3-DIOL |
| NHE | 2-[N-CYCLOHEXYLAMINO]ETHANE SULFONIC ACID |
| XPE | DECAETHYLENE GLYCOL |
| HEZ | HEXANE-1,6-DIOL |
| OES | N-OCTYL-2-HYDROXYETHYL SULFOXIDE |
| P6G | POLYETHYLENE GLYCOL PEG400 |
| DET | UNDECYLAMINE-N,N-DIMETHYL-N-OXIDE |
| 1PG | 2-(2-{2-[2-(2-METHOXY-ETHOXY)-ETHOXY]-ETHOXY}-ETHOXY)-ETHANOL |
| BOG | B-OCTYLGLUCOSIDE |
| HSG |  |
| SOG |  |
| HSH |  |
| SBY |  |
| LMT |  |
| 16A |  |
| LMU |  |
| CE1 |  |
| CAT |  |
| C15 |  |
| TCH |  |
| TBA |  |
| 4OA |  |
| PE4 | POLYETHYLENE GLYCOL PEG4000 |
| L |  |
| LI1 | 1-[2,6,10.14-TETRAMETHYL-HEXADECAN-16-YL]- 2-[2,10,14-TRIMETHYLHEXADECAN-16-YL]GLYCEROL |
